# Serologic IL-18 increase with B-cell IL-18R loss characterizes selective IgA deficiency

**DOI:** 10.3389/fimmu.2025.1687720

**Published:** 2026-01-05

**Authors:** Andri L. Lemarquis, Hildur Sigurgrimsdottir, Fannar P. Theodors, Ida Karnsund, Helga K. Einarsdottir, Gudmundur Jorgensen, Olov Ekwall, Ingileif Jonsdottir, Bjorn R. Ludviksson

**Affiliations:** 1 Department of Immunology, Landspítali-University Hospital, Reykjavík, Iceland; 2 Faculty of Medicine, University of Iceland, Reykjavík, Iceland; 3 Department of Pediatrics, Institute of Clinical Sciences, The Sahlgrenska Academy, University of Gothenburg, Gothenburg, Sweden; 4 Beckman Research Institute, City of Hope, Duarte, CA, United States; 5 Department of Rheumatology and Inflammation Research, Institute of Medicine, The Sahlgrenska Academy, University of Gothenburg, Gothenburg, Sweden; 6 deCODE Genetics, Division of Infectious and Inflammatory Diseases, Reykjavík, Iceland

**Keywords:** IgA, primary antibody deficiencies, IL18, TSLP, CpG, TWEAK, sCD40L, CCL3

## Abstract

**Introduction:**

Selective IgA deficiency (sIgAD) is the most common primary antibody deficiency in Western populations and is associated with increased risks of respiratory infections, atopy, and autoimmunity. However, the serologic and B-cell–intrinsic pathways underlying this immune dysregulation remain poorly defined. We sought to characterize a population-based adult sIgAD cohort clinically and immunologically and to identify soluble and transcriptional signatures that link cytokine milieu to B-cell dysfunction.

**Methods:**

We studied 61 adults with sIgAD and 73 age- and sex-matched healthy controls from the Icelandic sIgAD cohort. Participants completed standardized questionnaires on infections, atopy, and autoimmune disease. Serum immunoglobulins and autoantibodies (ANA, ENA, RF, CCP) were quantified, and a 65-plex Luminex cytokine/chemokine panel was measured in a subset of sIgAD individuals without active inflammatory disease and healthy controls. Purified CD19⁺ B cells from adults with sIgAD and controls were profiled by bulk RNA-seq at baseline and after 48 h TLR9 stimulation with CpG ODN 2006. Unsupervised analyses, differential expression, and correlation networks integrated clinical, serologic, and transcriptional data.

**Results:**

Clinically, adults with sIgAD had an airway-predominant infectious burden (notably increased sinusitis and pneumonia), a skin-skewed atopic pattern (eczema and urticaria), and frequent ANA/ENA positivity despite normal IgG and IgM. Immunoglobulin measurements showed very low IgA, increased total IgG and IgG1, and selectively reduced IgG4. Serum profiling revealed a coherent five-analyte signature, IL-18, sCD40L, TSLP, CCL3, and TWEAK, elevated in sIgAD and associated with higher IgG, lower residual IgA, and ANA/ENA positivity. This pattern was not reproduced in CpG-stimulated B-cell supernatants, indicating a non–B-cell origin. RNA-seq of purified B cells demonstrated diagnosis-dependent transcriptional programs at baseline and after CpG, with prominently reduced expression of IL-18 receptor components in sIgAD B cells.

**Discussion:**

Adult sIgAD is characterized by a blood-measurable endotype in which a systemic IL-18–centered soluble signature coexists with reduced IL-18 receptor expression and altered signaling programs in B cells. This IL-18-IL-18R axis aligns with ANA/ENA-associated immune dysregulation and an IgG-skewed class-switch profile, nominating IL-18–related mediators and B-cell IL-18R expression as candidate biomarkers and mechanistic targets in a subset of adults with sIgAD.

## Introduction

Selective Immunoglobulin A deficiency (sIgAD) is the most common primary antibody deficiency in the western world (1–3) to 1:3229 in Chinese and 1:18,500 in Japanese individuals (4, 5). It is classified as IgA ≤ 0,07g/L with normal concentrations of IgG and IgM. Individuals with sIgAD have a manyfold increased risk of developing immune mediated diseases such as autoimmunity, infections and atopic diseases (3). The molecular and genetic defects responsible are still largely unknown for the majority of individuals with hypogammaglobulinemias (6) and while these may be polygenic or epigenetic (7) phenotypic understanding of patients is needed. Especially due to the heterogenicity of the clinical manifestations (8), where better immunophenotyping of affected individuals could lead to individualized treatment options (9). Cellular phenotyping has demonstrated a defect enhanced by TLR9 stimulation in transitional B cells in sIgAD, pointing towards an early B-cell defect linked to T-cell independent B-cell responses (10) and phosphorylation disturbances in STAT3 (11). Furthermore, loss of tolerance has been demonstrated through the high prevalence of autoantibody (AAb) positivity in sIgAD, but no links to the affected underlying immunological pathways has been demonstrated (12, 13).

While considerable efforts have been done finding the gene/s responsible for the defect, no study has assessed the transcriptomic signatures of B cells from sIgAD individuals. The aims of this study were to evaluate the clinical phenotype of sIgAD in a whole population setting, with a randomized control group, and perform serological bio-typing and transcriptomic profiling of sIgAD. This is important since estimating the immune pathways responsible for sIgAD may be difficult in individuals already suffering from various associated comorbidities caused by immune dysregulation (14).

## Results

### The clinical phenotype of sIgAD individuals

Out of the 136 individuals previously identified in the Icelandic sIgAD cohort, previously described through screening and clinical diagnosis (2, 3), 100 were alive and 31 were under 18 years of age. Of the 69 adults, 61 (88%) accepted to participated and completed a detailed health-related questionnaire. Overall, adults with sIgAD reported a clear, clinically meaningful burden of disease compared to a randomly selected and age matched cohort (2).

Respiratory infections dominated the clinical picture. In the upper airways, recurrent sinus disease was the clearest discriminator between groups, whereas common viral colds, pharyngitis, laryngitis, and acute otitis media were broadly comparable. In the lower airways, pneumonias were notably more frequent in sIgAD, while bronchitis showed a milder difference. Many participants reported multiple antibiotic courses within the prior year. In contrast, gastrointestinal, urogenital, and skin infections did not differ meaningfully between groups, indicating that the excess infectious morbidity is concentrated in the respiratory tract (**Table 1**).

**Table 1:** Infectious, autoimmune, atopic, antibody and autoantibody profiling in sIgAD individuals.

Infections	HCs		SIgAD			
	Mean	SD	Mean	SD	p value	
Age	48.7	15.10	49.9	14.90	0.84	Ns
Upper respiratory tract infections	n	%	n	%	p	
Infectious conjunctivitis	21	27%	20	31%	0.7002	n.s.
Common viral cold (1 year)	63	86%	49	79%	0.3582	n.s.
Pharyngitis	56	76%	20	31%	0.4338	n.s.
Laryngitis	16	20%	20	31%	0.1596	n.s.
Sinusitis	26	34%	56	90%	0.0015	**
Acute otitis media	26	34%	7	10%	0.0792	n.s.
Lower respiratory tract infections	n	%	n	%	p	
Bronchitis SIgAD controls	23	30%	26	41%	0.2014	n.s.
Pneumonia	11	13%	21	33%	<0,0001	****
Gastrointestinal infections	n	%	n	%	p	
Campylobacter	11	13%	7	10%	0.7845	n.s.
Salmonella	11	13%	4	5%	0.1416	n.s.
Infectious gastroenteritis nos	13	15%	9	13%	0.8057	n.s.
Protozoal- or worm infections SIgAD	10	11%	15	23%	0.1006	n.s.
Urogenital infections	n	%	n	%	p	
Cystitis	14	17%	20	31%	0.0652	n.s.
Pyelonephritis	12	14%	8	11%	0.796	n.s.
Skin infections	n	%	n	%	p	
Bacterial skin infections	22	28%	15	23%	0.7002	n.s.

Comparison of self-reported infectious outcomes in adults with sIgAD (n=61) and age-matched healthy controls (HC; n=73). Data are n (%) unless otherwise indicated. Age is presented as mean ± SD. p values are two-sided (categorical outcomes by Fisher’s exact test; age by unpaired t test). Significance codes: n.s., not significant; *p<0.05; **p<0.01; ***p<0.001; ****p<0.0001. “nos” = not otherwise specified. Outcomes refer to the prior 12 months unless stated otherwise (e.g., “Common viral cold (1 year)”). Results indicate an airway-focused burden in sIgAD, with sinusitis and pneumonia showing the clearest between-group differences, while gastrointestinal, urogenital, and skin infections were broadly similar.

Atopy was prominent but selective. General eczema and allergic urticaria were clearly enriched, whereas asthma and allergic rhinitis/conjunctivitis were similar to controls; food allergy and anaphylaxis were uncommon in both groups. Autoimmune diagnoses were also represented, spanning organ-specific (e.g., thyroid) and systemic entities (**Table 2**). Hospitalizations for infection occurred but were relatively uncommon overall.

**Table 2:** Atopic profiling in sIgAD individuals.

Atopic diseases	HCs		sIgAD		p value	
	n	%	n	%		
Asthma	8	13%	9	15%	0.4338	n.s.
Allergic rhino conjunctivitis	12	19%	9	15%	0.1596	n.s.
Allergic urticaria	1	2%	5	8%	0.0015	**
Food allergy	1	2%	3	5%	0.0792	n.s.
Allergic anaphylaxis	0	0%	3	5%	0.2014	n.s.
General eczema	3	5%	16	26%	<0,0001	****
Contact dermatitis	0	0%	6	10%	0.7002	n.s.

Self-reported, physician-diagnosed atopic conditions in adults with sIgAD (n=61) and age-matched healthy controls (HC; n=73). Data are n (%). p values are two-sided and computed with Fisher’s exact test. Significance codes: n.s., not significant; *p<0.05; **p<0.01; ***p<0.001; ****p<0.0001. “Allergic rhinoconjunctivitis” denotes hay fever; “general eczema” includes atopic dermatitis/eczema; “allergic anaphylaxis” indicates any prior systemic allergic reaction meeting clinical criteria. Results indicate a skin-predominant atopic pattern in sIgAD, with enrichment of eczema and allergic urticaria, while asthma and rhinoconjunctivitis are comparable to controls.

To evaluate serologic autoimmunity independent of clinical autoimmune disease, we examined adults with sIgAD who had no prior autoimmune diagnoses. Even in this subset, autoantibody positivity was frequent compared with controls. The signal was driven largely by antinuclear antibodies (ANA) and extractable nuclear antigens (ENA); in contrast, isolated rheumatoid factor (RF) and anti-CCP did not differ meaningfully (**Table 3**).

**Table 3:** Autoimmune and serologic profiling in sIgAD individuals.

	HCs		sIgAD		p value	
Autoimmunity	3	4%	12	16%	0.0031	**
Autoantibodies	n	%	n	%	p	
ANA	0	0%	8	28%	0.0021	**
ENA	0	0%	5	17%	0.0336	*
RF	4	11%	5	17%	1	Ns
CCP	3	9%	5	17%	0.2446	Ns
Antibody classes	n	%	n	%	p	
IgM	0.93	0.44	0.93	0.43	0.850	Ns
IgA	2.22	0.78	0.05	0.03	< 0.0001	****
IgG	10.62	2.33	13.66	3.33	0.0004	***
IgG1	7.76	1.95	10.59	3.08	0.0004	***
IgG2	3.53	1.60	3.66	1.54	0.776	Ns
IgG3	0.39	0.23	0.44	0.29	0.516	Ns
IgG4	0.50	0.42	0.22	0.21	0.0008	***

Comparison of self-reported data regarding autoimmunity in 61 individuals with selective IgA deficiency (sIgAD) and 73 matched controls. For systemic presentations one had psoriasis, two had rheumatoid arthritis, one had sarcoidosis and one had polymyalgia rheumatica. For organ specific autoimmunity two had primary hypothyroidism one had pernicious anemia, one had pemphigus, one with PLEVA skin disease (Pityriasis lichenoides et varioliformis acuta), one had interstitial cystitis, one had Multiple Sclerosis and one had pemphigus. Data are n (%) for categorical variables and mean ± SD for immunoglobulins. p values are two-sided (Fisher’s exact for categorical; unpaired t test for continuous). Significance codes: n.s., not significant; *p<0.05; **p<0.01; ***p<0.001; ****p<0.0001.

In the same cohort, the immunoglobulin profile paralleled these serologic findings. As expected, IgA was markedly reduced. Total IgG was higher, driven primarily by IgG1, whereas IgG2 and IgG3 were comparable to controls. By contrast, IgG4 was selectively lower. This pattern, very low IgA with increased IgG/IgG1 and reduced IgG4, provides context for the ANA/ENA-centered autoreactivity and supports altered class-switching dynamics in adult sIgAD.

### sIgAD individuals have a dysregulated cytokine profile in serum compared to HC

**Figure 1:**
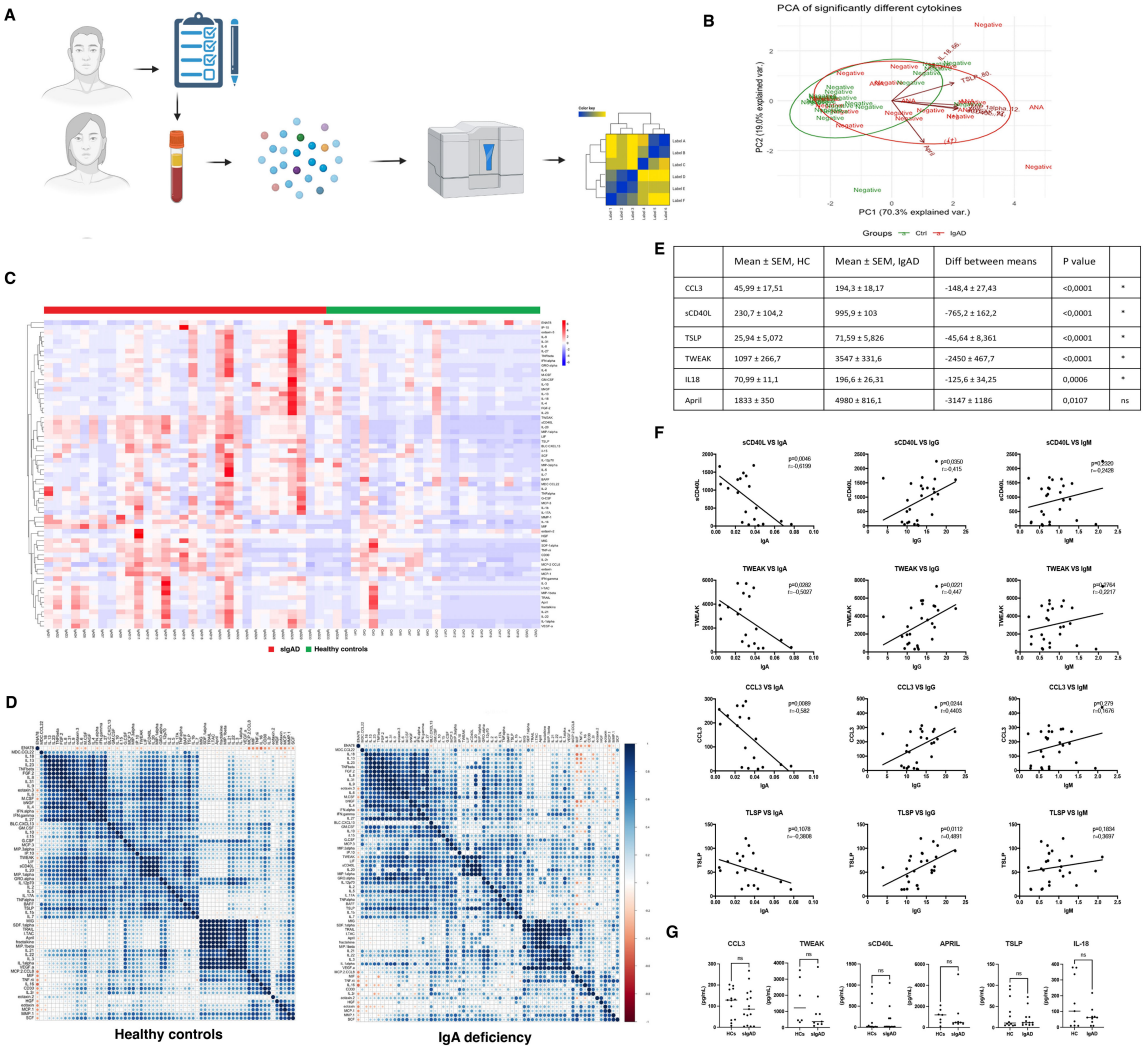
A serological immune dysregulation linked to autoantibody production is seen in sIgAD individuals. **(A)** Cohort and workflow. Adults with sIgAD (N = 61) and age-matched healthy controls (N = 73) completed clinical questionnaires and provided serum and PBMCs. Serum was profiled on a 65-plex cytokine/chemokine panel (Luminex). Immunoglobulins and autoantibodies were measured; a subset contributed purified B cells for *in-vitro* assays and RNA-seq (**Figure 2**). **(B)** Principal component analysis (PCA) of autoscaled analyte concentrations, sIgAD (red) and HC (green). ANA-positive sIgAD samples are highlighted. Axes indicate variance explained by PC1/PC2; 95% confidence ellipses are shown. ANA+ vs ANA- sIgAD individuals and their representative concentration of elevated factors. **(C)** Heat map of serum factors. Z-scored concentrations for a representative subset of analytes illustrate the broader pattern of dysregulation in sIgAD versus HC. Rows are clustered; column annotations indicate group and ANA status. **(D)** Reorganized correlation network in sIgAD. Clustered Spearman correlation matrices visualize pairwise relationships among the 65 analytes in sIgAD and HC. Color encodes direction (positive/negative), and symbol size/opacity reflects magnitude/significance. sIgAD displays tighter co-variation among select mediators. **(E)** Differentially abundant mediators for the five analytes elevated in sIgAD after family-wise correction, IL-18, sCD40L, TSLP, CCL3, and TWEAK, are shown per individual. Two-sided tests with Bonferroni correction across 65 analytes; adjusted p values appear in the panel note (see **Table 1** for adjusted p-values). **(F)** Associations between the five-analyte signature and immunoglobulin classes show that higher IL-18/sCD40L/TSLP/CCL3/TWEAK levels track with total IgG and relate inversely to the small residual IgA detectable in many sIgAD individuals. Scatter plots with linear fits are shown; correlation coefficients and multiplicity-adjusted p values are reported in the panel note. (H(C)) CpG-stimulated B-cell supernatants. **(G)** Concentrations of the same mediators measured in supernatants after 48 h TLR9 stimulation (CpG ODN 2006) of purified B cells show no consistent group differences after multiple-testing correction, suggesting that the *in-vivo* serum signature is not reproduced by isolated B-cell stimulation under these conditions. Multiple-testing control uses Bonferroni for the 65-plex and Benjamini–Hochberg for correlation matrices unless otherwise specified. Exact n, test details, and thresholds are provided in panel notes and Methods. sIgAD, selective IgA deficiency; HC, healthy control; ANA, antinuclear antibody; APRIL, TNFSF13.

To assess the serological drivers of the observed clinical and serologic alterations in sIgAD, we profiled a 65-analyte cytokine/chemokine panel in serum from adults with sIgAD and age-matched healthy controls and integrated these data with immunoglobulin measurements and autoantibody status (**Figure 1A**). At a global level, unsupervised analyses revealed a reorganized soluble mediator landscape in sIgAD. In principal-component space (15), samples separated by group, indicating coordinated shifts across multiple pathways rather than isolated changes in single analytes (**Figures 1B**). This systems-level view was reinforced by the correlation structure: in sIgAD, cytokines and chemokines clustered into tighter modules than in controls, with a prominent block containing IL-18, sCD40L, TSLP, CCL3, and TWEAK (**Figures 1C, D**). Focusing on individual mediators, these five factors were consistently higher in sIgAD after family-wise correction (**Figure 1E**). Several additional analytes (including APRIL, VEGF-A, IL-20, and IL-22) shifted in the same direction but did not meet the predefined multiple-testing threshold, suggesting broader pathway engagement beyond the core signature. Strikingly, these soluble factors tracked positively with total IgG and inversely with the small residual IgA that remains detectable in many sIgAD individuals (**Figure 1F**) indicating potential compensatory mechanisms to increase IgA production. This suggests a link between autoantibody production and the concentrations of these factors. However, when isolated B cells from sIgAD were stimulated with CpG for 48h, no significant differences were found in the concentrations of these factors in the supernatant (**Figure 1G**), indicating a B cell extrinsic origin for these soluble factors.

### The transcriptomic profiles of sIgAD individuals link to serologic immune imbalances and to signal transduction

**Figure 2:**
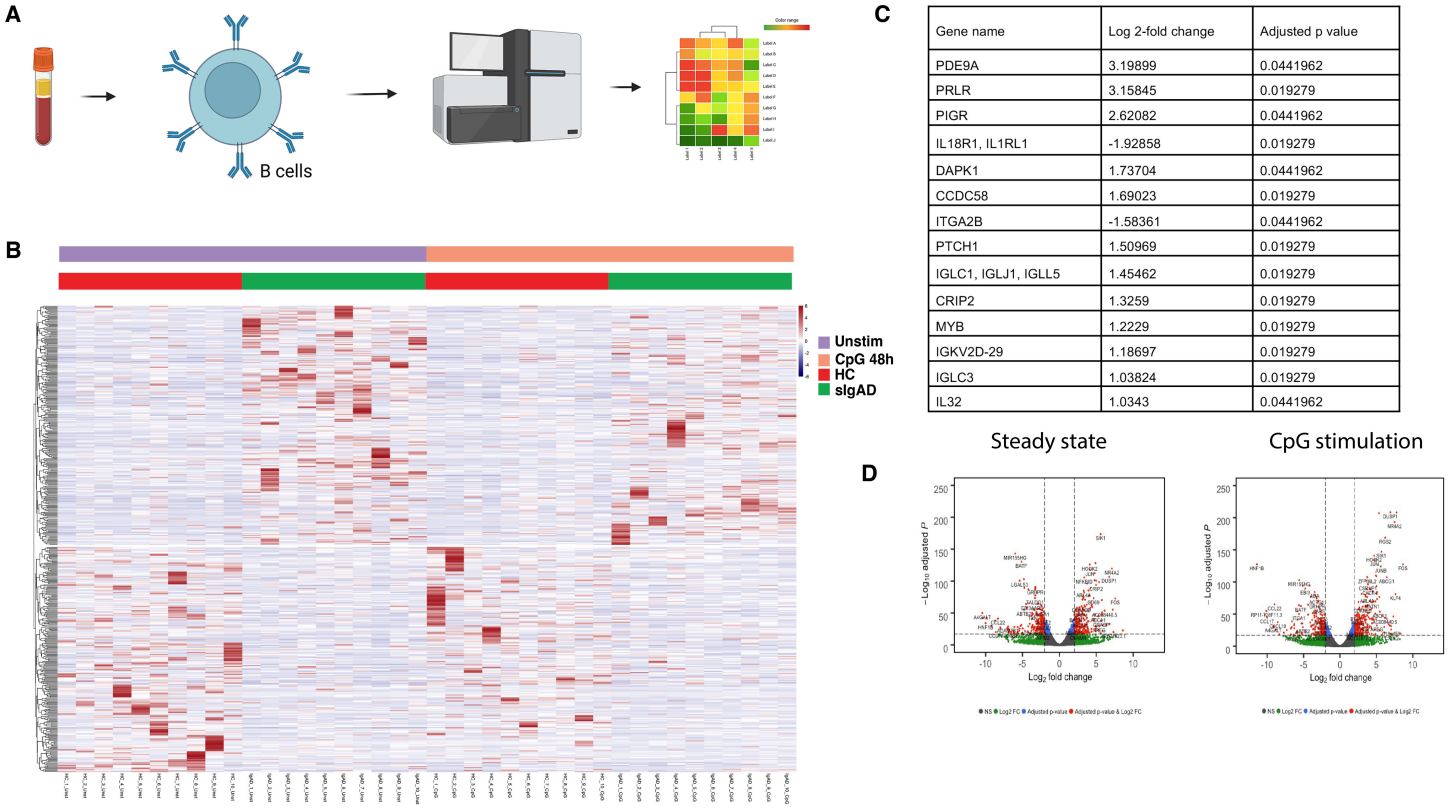
Transcriptomic B cell alterations in selective IgA deficiency. **(A)** Study overview and workflow. Purified CD19^+^ B cells from adults with sIgAD (n=10) and age-/sex-matched healthy controls (HC; n=10) were profiled at baseline and after 48 h TLR9 activation with CpG ODN 2006 (1 µg/ml TLR9 agonist, InvivoGen). RNA was extracted for bulk RNA-seq and integrated with serum findings from **Figure 1**. **(B)** Sample-level structure across conditions. Heat map of variance-stabilized expression (row-scaled) for high-variance genes shows separation by diagnosis and by stimulation state (± CpG). Columns are clustered by hierarchical clustering (Euclidean distance, Ward.D2); rows by correlation distance. Group and stimulation annotations are shown above. **(C)** Top differentially expressed transcripts highlight receptor-proximal changes. Ranked list (or bar plot) of the most strongly and consistently altered genes between sIgAD and HC at baseline (pre-CpG), emphasizing reduced expression of the IL-18 receptor components IL18R1 and IL18RAP in sIgAD B cells. Values shown are effect sizes from the DE model; FDR control is Benjamini–Hochberg (thresholds in Methods/panel note). **(D)** Genome-wide differential expression overview. Volcano representation of sIgAD vs HC at baseline (and, if shown, after CpG) with genes passing predefined |log_2_FC| and FDR thresholds highlighted. Key transcripts (including IL18R1/IL18RAP) are labeled for orientation. Counts were normalized and variance-stabilized prior to unsupervised analyses. Multiple-testing correction used Benjamini–Hochberg unless otherwise indicated. Exact n per group/condition, DE thresholds (|log_2_FC|, FDR), clustering/linkage settings, and software versions are provided in panel notes and Methods. Gene symbols refer to human HGNC nomenclature; in the main text, we refer to the protein as IL-18R and the transcripts as IL18R1/IL18RAP.

Because the five-analyte serum signature (IL-18, sCD40L, TSLP, CCL3, TWEAK) was not reproduced by isolated B cells after CpG stimulation, we next asked whether this milieu nevertheless imprints B cells and through what mechanisms (**Figure 2A**). We profiled purified CD19^+^ B cells from adults with sIgAD without overt clinical symptoms of autoimmunity, to remove any confounding effects of secondary inflammation, and matched controls at baseline and after TLR9 activation. Unsupervised sample-level structure separated sIgAD from controls and distinguished unstimulated from CpG-stimulated states, indicating that B-cell transcriptional programs differ at baseline and remain divergent under activation (**Figure 2B**). At the gene level, differences mapped to pathways relevant for activation and class switching, rather than to broad cell-identity shifts (**Figure 2C**).

An important transcriptional feature in sIgAD B cells was reduced expression of the IL-18 receptor components IL18R1 and IL18RAP (**Figure 2D**), which has been associated with self reactive antibody responses (16). This finding links the elevated systemic IL-18 observed in serum to a receptor-proximal change in B cells, consistent with diminished capacity to sense or transduce IL-18 signals. The reduction was evident at baseline and persisted after CpG, suggesting a stable trait rather than a transient activation effect.

## Discussion

We combined clinical phenotyping, targeted serum profiling, and B-cell transcriptomics to define an immune endotype of adult selective IgA deficiency (sIgAD). Clinically, morbidity was airway-predominant (recurrent sinus disease and increased pneumonias), with a skin-biased atopic pattern (eczema and urticaria) and frequent ANA/ENA positivity even in individuals without diagnosed autoimmune disease. Serologically, we identified a coherent five-analyte signature, IL-18, sCD40L, TSLP, CCL3, and TWEAK, elevated in sIgAD after family-wise correction. Transcriptomically, purified CD19^+^ B cells from sIgAD showed reduced expression of IL-18 receptor components (IL18R1 and IL18RAP) at baseline and after TLR9 activation, alongside pathway-level shifts in signal-transduction programs. Together, these findings outline a blood-measurable endotype in which a systemic IL-18 centered phenotype with diminished transcriptional expression of IL-18 receptor expression in B-cells.

Some PADs may be more closely linked to certain infections, autoimmunity, atopy or malignancy depending on their mechanisms (9, 17, 18). Regarding sIgAD individuals their phenotype is heterogeneous, with some individuals remaining asymptomatic as the first examples described, while others suffer from various comorbidities. A classification scheme based on the literature has been proposed for clinicians to assess sIgAD (19). Based on that sIgAD could be classified into the following groups: a) largely asymptomatic individuals, b) these with minor infections, c) atopic diseases, d) autoimmunity and finally e) sIgAD with severe concurrent complications. Thus, reflecting everything from a relatively benign course to individuals suffering from severe diseases and double mortality rate compared with the general population (20, 21).

Prior studies have established that adults with sIgAD do carry increased risks of respiratory infections, atopy, and autoimmunity (22–24), and that B-cell signaling can be perturbed under defined stimuli. Our data extend this literature in three ways. First, we show that the serologic phenotype is not diffuse: a concentrated set of soluble mediators, IL-18, sCD40L, TSLP, CCL3, and TWEAK, are dysregulated in sIgAD. Second, by linking this serum signature to reduced IL18R1/IL18RAP expression in B cells, we provide a receptor-proximal foothold connecting the circulating milieu to B-cell state. Third, we integrated these layers with immunoglobulin class measurements. In these measurements we observed that very low IgA (<0.07g/L) coincided with higher total IgG(g/L). This was mainly driven by IgG1 (g/L), and selectively reduced IgG4 (g/L), providing indications of broader B cell dysregulation in sIgAD patients.

The non–B-cell origin of the serum signature although not B cell derived could have multiple direct or indirect sources. T cell help might be responsible, where TSLP is well known to play roles in thymic function, and as such thymic homeostasis may contribute (25–27). Myeloid and activated T cells can secrete CCL3 and TWEAK, coordinating recruitment and costimulation in inflamed tissues (28). The serologic IL-18 rise coupled to reduced B-cell IL-18R (IL18R1/IL18RAP) expression due to chronic upstream activation and receptor down-tuning on B cells. Platelet activation is a well-established source of sCD40L, fitting a non–B-cell origin and linking systemic inflammation or tissue damage to GC-adjacent signals (29). Together, these etiologies may explain why CpG-stimulated B-cell supernatants did not recapitulate the serum pattern and suggest a model in myeloid or primary lymphoid drives an IL-18–centered milieu that secondarily affects B cells.

IL-18 is a pleiotropic cytokine best known for amplifying type-1 responses in concert with IL-12, but it can also shape humoral immunity indirectly through effects on accessory cells and cytokine networks (16, 22, 30, 31). In our cohort, IL-18 rose alongside factors linked to germinal-center (GC) function or B-cell help (sCD40L, CCL3, TSLP, TWEAK), and these mediators co-varied with higher IgG and lower residual IgA (4, 10). Although we did not localize the cellular sources, the absence of group differences in CpG-stimulated B-cell supernatants supports a non–B-cell origin for the serum signature under our assay conditions. Platelets, T cells, myeloid cells, stromal/epithelial compartments, or tissue inflammation could plausibly contribute and merit future work. Although this study does not have orthogonal validation methods to assess IL18R expression in B cells, further studies are needed to address this looking at transcriptional or protein expression at a higher resolution.

A notable feature is the simultaneous elevation of IL-18 in serum and reduction of IL-18 receptor expression on B cells. We interpret this conservatively as a state of diminished B-cell IL-18 sensing in the context of increased systemic IL-18. Whether this reflects adaptive down-regulation from chronic cytokine exposure, cell-intrinsic transcriptional regulation, or differences in B-cell subset composition cannot be resolved here. Functionally, lower IL18R1/IL18RAP expression could blunt IL-18–dependent costimulation during B-cell activation, potentially altering selection thresholds or antibody output in GC-related contexts.

Within sIgAD, ANA/ENA aligned with the multidimensional axes dominated by the five signature mediators and with higher values on the PCA axis aligning ANA with serum associated changes in sIgAD patients. However out of the four top altered factors in sIgAD patients, only TLSP was significantly altered between ANA positive and negative individuals. Altogether that the cytokine altered milieu may track with serologic alterations. The immunoglobulin pattern—very low IgA, increased IgG/IgG1, and lower IgG4, further situates the endotype within altered class-switch dynamics. While we cannot assign causality, these converging observations are consistent with a model in which chronic innate/adjuvant-like cues (captured by IL-18 and companion mediators) shape B-cell activation in a way that favors autoreactivity in a subset of adults with sIgAD.

At the same time, atopy in sIgAD was selective rather than global: eczema and urticaria were enriched, whereas asthma and rhinoconjunctivitis were comparable to controls. The serum signature we identify is not a generic Th2 readout; instead, it centers on mediators with GC and tissue-repair/immunoregulatory roles. This distinction may help explain why atopy in sIgAD skews to the skin rather than the airways, despite overall airway-predominant infectious morbidity. While studies have previously characterized smaller numbers of individuals in the Icelandic sIgAD cohort, the present study does not have the power to accurately track symptom emergence longitudinally, however our findings underscore the need for long-term follow-up in larger cohorts (2, 3).

The five-analyte (IL-18, sCD40L, TSLP, CCL3, TWEAK) and the composite score derived from it are measurable in serum and could be used to stratify adults with sIgAD, particularly when combined with ANA/ENA testing. Such stratification might help identify individuals at higher risk of immune dysregulation who warrant closer follow-up or targeted counseling. Mechanistically, the convergence on an IL-18–centered signature, together with reduced B-cell IL-18R expression—supports hypothesis-driven exploration of the IL-1/IL-18 axis and B-cell–directed approaches in ANA-positive sIgAD. We emphasize that our data are observational; therapeutic implications remain exploratory and will require prospective evaluation.

While this study is population based, it has a modest sample size for the serum and RNA-seq components. Clinical data include retrospective questionnaire elements and may under- or over-estimate events. The design is cross-sectional, precluding causal inference or temporal ordering of cytokine changes, autoantibody development, and clinical outcomes. We did not perform mucosal sampling, which limits insight into tissue sources of the mediators. B-cell RNA-seq was bulk rather than single-cell, so subset-specific changes could be missed. Finally, we did not test IL-18 co-stimulation in functional B-cell assays, which would directly probe consequences of reduced IL-18R expression.

CD40 has been reported to be suggestively associated with Swedish sIgAD patients carrying HLA-B*08:01-DRB1*03:01-DQB1*02:01. In future studies it would be interesting to assess if carrying this specific haplotype is related to the immunological parameters analyzed. Especially since IL1R1, which is in very close proximity to IL1RL1 and IL18R1 on chromosome 2, has been previously reported associated with Swedish IgAD carrying HLA-DRB1*07:01-DQB1*02:02 (32, 33). Thus, it would be valuable to investigate whether individuals in the B cells transcriptomic cohort carrying this MHC haplotype, and whether any potential correlation exists.

Adult sIgAD exhibits a reproducible soluble mediator signature centered on IL-18, coupled to reduced B-cell IL-18 receptor expression and altered signaling programs. This endotype aligns with ANA/ENA positivity and provides a coherent, blood-measurable framework for understanding immune dysregulation beyond the defining IgA deficiency. The findings nominate testable biomarkers and mechanisms to pursue in prospective studies and, ultimately, in pathway-informed therapeutic strategies.

## Materials and methods

### Demographics of the study group

61 IgA deficient individuals and 73 age and sex matched controls were included for the analysis of the clinical phenotype. Of these 24 individuals without active inflammation and 21 HCs were assessed for serological measurements. The sIgAD individuals were found through three ways as prior described (2). Shortly, by screening 4004 blood donors for sIgAD using serum samples that were collected at the Icelandic blood bank during 1999-2001. Seven blood donors were found to have sIgAD, and 5 consented to participate in the study and their sIgAD status was confirmed by new serum analyses, (II) By re-evaluating sIgAD individuals from a previous sIgAD study (1974–1979) in Iceland. Originally, 26 sIgAD individuals had been identified by screening of 15,663 individuals, of which 13,942 were blood donors (34) Eleven individuals fulfilled the study criteria for sIgAD (serum IgA < 0.07 g/L) and were recruited in the study and (III) by evaluating individuals that were identified during the time period 1992–2017 to have low serum IgA levels when analyzed at the Departments of Clinical Chemistry and Immunology, Landspitali – The National University Hospital of Iceland (LSH-CCID). All of these were collected together through the deCode genetics database. All 61 individual answered a clinical questionnaire as previously described. Peripheral blood from 24 IgA deficient individuals (median age 48,7± SD 15.10 years) from the Icelandic sIgAD group, that had not active infection, autoimmunity or serious atopic manifestations, where collected into heparinized tubes after obtaining informed consent (in accordance with procedures approved by The National Bioethics Committee and The Data Protection Authority in Iceland). These were compared to gender and age matched healthy controls (median age 49.9 median age ± SD 14.90 years).

### Autoantibody quantification

For the determination of IgG antibodies to Cyclic Citrullinated Peptides (CCP) in human sera the Immunoscan CCplus^®^ ELISA test kit was performed according to the instructions of the manufacturer (Euro Diagnostica AB, Lundavägen 151, SE-212–24 Malmö, Sweden). The screening for Rheumatoid factor (RF) was performed using ELISA with rabbit IgG diluted (Thermo Fisher Scientific, MA, USA) and Monoclonal Anti-Human Kappa Light Chain (Sigma, St. Louis, MO, USA), Diluted Polyclonal Rabbit Anti-Mouse Immunoglobulins/AP (Dako, DK-2600 Glostrup, Denmark) ELISAs were analyzed with Microplate photometer Multiskan EX (Thermo Fisher Scientific, MA, USA).

For the isotyping of RF Monoclonal Anti-Human Kappa Light Chain (Sigma, St. Louis, MO, USA), Monoclonal Anti-Human IgM-Alkaline Phosphatase antibody produced in mouse (Sigma, St. Louis, MO, USA) and AKP Mouse Anti-Human IgA1/IgA2 (BD Pharmingen™, BD Biosciences, NJ, USA) were used. Antinuclear antibodies were analyzed as used in the clinic by indirect immunofluorescence (IIF) using rat tissue for non-organ-specific antibodies with Diluted Polyclonal Rabbit Anti-Human IgA, IgG, IgM, Kappa, Lambda/FITC (Dako, DK-2600 Glostrup, Denmark). The ANA was read with optical microscope DM 2500 LED (Leica Microsystems CMS GmbH, address). Positive samples were recognized as diffuse, speckled, membrane or nucleolar pattern. Antinuclear IgG antibodies in sera were detected by fluorescence enzyme immunoassay on the Phadia^®^ 250 system using EliA™ CTD screen (Thermo Fisher Scientific, MA, USA). The wells are coated with U1RNP (RNP70, A, C), SS-A/Ro52, SS-A/RO60, SS-B/La, Centromere B, Scl-70, Jo-1, fibrillarin, RNA Pol III, Rib-P, PM-Scl, PCNA, Mi-2 proteins, Sm proteins and native purified DNA. In addition to this screening assay, Phadia EliA™ assays were used to measure antibodies to individual antigens. Antibodies to individual antigens were determined in all individuals that tested positive with the EliA CTD screen assay. The assays were performed according to the instructions of the manufacturer.

### Measurement of IgG, IgA IgM by nephelometry and RID

For measurements of serum IgA, IgG and IgM the IMMAGE^®^ 800 Immunochemistry System (Beckman Coulter, Inc., address) was used. Measurements of IgG-subclasses were carried out using radial immunodiffusion technique using procedures according to local protocol of the Department of Immunology, Landspítali University hospital, Iceland.

### Luminex

Serum samples and supernatants from CpG stimulated B cells were analyzed with Luminex. Chemokines and cytokines were measured using a magnetic Immune Monitoring 65-Plex Human ProcartaPlex™ Luminex assay (R&D systems) and analyzed in Bio-Plex 200 system (Bio-Rad Laboratories, California, USA).

### Isolation of PBMCs, B cells and stimulation with CpG ODN 2006

Peripheral Blood Mononuclear cells (PBMCs) were isolated from heparinized peripheral blood using Ficoll–Paque gradient centrifugation (Sigma-Aldrich, St. Louis, MO, USA). CD19+ B cells were isolated by magnetic bead-based positive selection using Dynabeads and DETACHaBEAD CD19 (Invitrogen). All isolations were done according to the manufacturer’s instructions. The purity of isolated CD19+ B cells was consistently >95% as analyzed by flow cytometry.

### RNA sampling and processing

TotalRNA was isolated from 40 samples using RNeasy micro kit (Qiagen Inc. (cat. no. 74004)) according to the manufacturer’s instructions (including a DNAse treatment). 10 samples where unstimulated B cells from HC, 10 samples unstimulated B cells from sIgAD individuals, 10 sample CpG stimulated B cells from HC and 10 sample CpG stimulated B cells from sIgAD individuals. RNA was eluted with 14 uL RNAse free water and stored at -80 degrees Celsius. We quantified and QC-ed the RNA samples by qPCR using Exilerate LNATM qPCR cDNA synthesis kit from Exiqon AS (Cat no.303301) and an endogenous control qPCR assay for the housekeeping gene GAPDH (Cat No 308005) and ACTB (β-actin) (Cat.no.308001). The amplification was performed in a LightCycler^®^ 480 Real-Time PCR System (Roche) in 384 well plates. The amplification curves were analyzed using the Roche LC software, for the determination of Cq (by the 2nd derivative method). We used 2 μL of the total RNA in the cDNA synthesis and diluted the cDNA 10fold in water before performing qPCR in duplicate per sample.

### Library preparation and next generation sequencing

The double stranded cDNA synthesis was performed using the SMARTer^®^ UltraTM Low Input RNA Kit for Sequencing (Cat. No. 634891, Clontech Laboratories, version 4) as by the manufacturer using 12 cycles of pre-PCR cDNA amplification. The cDNA was purified using AmpureXP beads using 1.8 x bead volume and washed twice in 80% EtOH. cDNA was re-suspended in 17 uL water (Beckman Coulter). The cDNA size distribution was validated, and quality inspected on a Bioanalyzer high sensitivity DNA chip (Agilent Technologies). The size distribution analysis showed a smear of about 2000 – 9–000 bp in size (peaked at approx. 2500–3000 bp). Samples were quantified by calculating the concentration on the bioanalyzer/Tapestation (size range 500–8000 bp.) using the Smear Analysis feature.

### NGS library generation and sequencing

The libraries made using the NexteraXT transposon-based library generation kit (Illumina Inc.) using 250 pg input material from the cDNA amplification, according to the manufacturer’s instructions (see attached protocol). The DNA was post-PCRed for 12 cycles and purified using AmpureXP beads as described above and eluated in 30 uL water. Finally, the libraries size distribution was validated, and quality inspected on a Bioanalyzer high sensitivity DNA chip (Agilent Technologies). The library peak is broad in the size range between 200–500 bp (and peaked at approx. 350 bp). The Nextera libraries were quantified by calculating the concentration on the bioanalyzer (size range 150–1000 bp) using the Smear Analysis feature and pooled accordingly. The library pool was quantified with qPCR (KAPA qPCR library quantification kit) and optimal concentration of the library pool used to generate the clusters on the surface of NextSeq500 flowcells (4 flowcells/4 nextseq runs) before sequencing using v2 chemistry according to the manufacturer instructions (Illumina Inc.) using Paired End 2x51 specifications.

### Pathway analysis

To study how stimulation with CpG affects B cells and which pathways are differentially expressed between sIgAD individuals and HCs, GO gene set enrichment and Ingenuity Pathway Analysis (IPA, Redwood City, California, USA; http://www.ingenuity.com/), a knowledge-based software package, was used.

### Statistical analysis

Statistical analyses and graphs were made using GraphPad Prism (V 8.0.1 GraphPad Software Inc, for windows, La Jolla, CA, USA) or R version 4.0.3 with RStudio Desktop version 1.4.1103. For cellular testing ANOVA was used for multiple comparisons, Mann-Whitney or Student’s t-test when the data was normally distributed for smaller groups. Results are expressed as mean ± SEM. Adjusted P values (Bonferroni correction) <0.05 were considered significant. The chi-squared or the Fisher’s exact tests were applied for categorical variables. Correlations between continuous variables were assessed with Pearson’s or Spearman’s correlation coefficients depending on normality of the data. The correlation matrix of serum factors and immunoglobulins was displayed using the R package corrplot; each factor is arranged by the angular order of their correlation matrix eigenvectors which places similar variables adjacently.

## Data Availability

All anonymized data underlying the results of this study are provided in the **Supplementary Material**. Any additional requirements of data will be made freely available on reasonable request to the corresponding authors.

## References

[1] Al-Herz W Bousfiha A Casanova JL Chapel H Conley ME Cunningham-Rundles C . Primary immunodeficiency diseases: an update on the classification from the international union of immunological societies expert committee for primary immunodeficiency. Front Immunol. (2011) 2:54. doi: 10.1007/s10875-015-0201-1, PMID: 22566844 PMC3342372

[2] Jorgensen GH Gardulf A Sigurdsson MI Sigurdardottir ST Thorsteinsdottir I Gudmundsson S . Clinical symptoms in adults with selective IgA deficiency: a case-control study. J Clin Immunol. (2013) 33:742–7. doi: 10.1007/s10875-012-9858-x, PMID: 23389234

[3] Jorgensen GH Thorsteinsdottir I Gudmundsson S Hammarstrom L Ludviksson BR . Familial aggregation of IgAD and autoimmunity. J Clin Immunol. (2009) 131:233–9. doi: 10.1016/j.clim.2008.11.013, PMID: 19167929

[4] Cunningham-Rundles C . Physiology of igA and igA deficiency. J Clin Immunol. (2001) 21:303–9. doi: 10.1023/A:1012241117984, PMID: 11720003

[5] Feng ML Zhao YL Shen T Huang H Yin B Liu RZ . Prevalence of immunoglobulin A deficiency in Chinese blood donors and evaluation of anaphylactic transfusion reaction risk. Transfusion Med (Oxford England). (2011) 21:338–43. doi: 10.1111/j.1365-3148.2011.01082.x, PMID: 21658139

[6] van der Burg M van Zelm MC Driessen GJ van Dongen JJ . New frontiers of primary antibody deficiencies. Cell Mol Life Sci. (2012) 69:59–73. doi: 10.1007/s00018-011-0836-x, PMID: 22042269 PMC11114824

[7] Jonsson S Sveinbjornsson G de Lapuente Portilla AL Swaminathan B Plomp R Dekkers G . Identification of sequence variants influencing immunoglobulin levels. Nat Genet. (2017) 49:1182. doi: 10.1038/ng.3897, PMID: 28628107

[8] Wang N Shen N Vyse TJ Anand V Gunnarson I Sturfelt G . Selective IgA deficiency in autoimmune diseases. Mol Med (Cambridge Mass). (2011) 17:1383–96. doi: 10.2119/molmed.2011.00195, PMID: 21826374 PMC3321806

[9] Cunningham-Rundles C . Autoimmunity in primary immune deficiency: taking lessons from our patients. Clin Exp Immunol. (2011) 164 Suppl 2:6–11. doi: 10.1111/j.1365-2249.2011.04388.x, PMID: 21466546 PMC3087904

[10] Lemarquis AL Einarsdottir HK Kristjansdottir RN Jonsdottir I Ludviksson BR . Transitional B cells and TLR9 responses are defective in selective igA deficiency. Front Immunol. (2018) 9. doi: 10.3389/fimmu.2018.00909, PMID: 29755476 PMC5934527

[11] Lemarquis AL Theodors F Einarsdottir HK Ludviksson BR . Mapping of signaling pathways linked to sIgAD reveals impaired IL-21 driven STAT3 B-cell activation. Front Immunol. (2019) 10. doi: 10.3389/fimmu.2019.00403, PMID: 30936864 PMC6431630

[12] Barka N Shen GQ Shoenfeld Y Alosachie IJ Gershwin ME Reyes H . Multireactive pattern of serum autoantibodies in asymptomatic individuals with immunoglobulin A deficiency. Clin Diagn Lab Immunol. (1995) 2:469–72. doi: 10.1128/cdli.2.4.469-472.1995, PMID: 7583926 PMC170181

[13] Swain S Selmi C Gershwin ME Teuber SS . The clinical implications of selective IgA deficiency. J Transl Autoimmun. (2019) 2:100025. doi: 10.1016/j.jtauto.2019.100025, PMID: 32743511 PMC7388344

[14] Cinicola BL Pulvirenti F Capponi M Bonetti M Brindisi G Gori A . Selective igA deficiency and allergy: A fresh look to an old story. Med (Kaunas). (2022) 58(1):129. doi: 10.3390/medicina58010129, PMID: 35056437 PMC8781177

[15] Evans MD Esnault S Denlinger LC Jarjour NN . Sputum cell IL-1 receptor expression level is a marker of airway neutrophilia and airflow obstruction in asthmatic patients. J Allergy Clin Immunol. (2018) 142:415–23. doi: 10.1016/j.jaci.2017.09.035, PMID: 29103994 PMC6019639

[16] Enoksson SL Grasset EK Hägglöf T Mattsson N Kaiser Y Gabrielsson S . The inflammatory cytokine IL-18 induces self-reactive innate antibody responses regulated by natural killer T cells. Proc Natl Acad Sci U S A. (2011) 108:E1399–407. doi: 10.1073/pnas.1107830108, PMID: 22135456 PMC3251096

[17] Liblau RS Bach JF . Selective IgA deficiency and autoimmunity. Int Arch Allergy Immunol. (1992) 99:16–27. doi: 10.1159/000236330, PMID: 1483063

[18] Schmidt RE Grimbacher B Witte T . Autoimmunity and primary immunodeficiency: two sides of the same coin? Nat Rev Rheumatol. (2017) 14:7–18. doi: 10.1038/nrrheum.2017.198, PMID: 29255211

[19] Yazdani R Latif A Tabassomi F Abolhassani H Azizi G Rezaei N . Clinical phenotype classification for selective immunoglobulin A deficiency. Expert Rev Clin Immunol. (2015) 11:1245–54. doi: 10.1586/1744666X.2015.1081565, PMID: 26306496

[20] Ludvigsson JF Neovius M Hammarstrom L . IgA deficiency & mortality: a population-based cohort study. J Clin Immunol. (2013) 33:1317–24. doi: 10.1007/s10875-013-9948-4, PMID: 24122027

[21] Mellemkjaer L Hammarstrom L Andersen V Yuen J Heilmann C Barington T . Cancer risk among patients with IgA deficiency or common variable immunodeficiency and their relatives: a combined Danish and Swedish study. Clin Exp Immunol. (2002) 130:495–500. doi: 10.1046/j.1365-2249.2002.02004.x, PMID: 12452841 PMC1906562

[22] Tsutsumi N Kimura T Arita K Ariyoshi M Ohnishi H Yamamoto T . The structural basis for receptor recognition of human interleukin-18. Nat Commun. (2014) 5:5340. doi: 10.1038/ncomms6340, PMID: 25500532 PMC4275594

[23] Jamee M Alaei MR Mesdaghi M Noorian S Moosavian M Dolatshahi E . The prevalence of selective and partial immunoglobulin A deficiency in patients with autoimmune polyendocrinopathy. Immunol Invest. (2022) 51:778–86. doi: 10.1080/08820139.2021.1872615, PMID: 33432864

[24] Odineal DD Gershwin ME . The epidemiology and clinical manifestations of autoimmunity in selective igA deficiency. Clin Rev Allergy Immunol. (2020) 58:107–33. doi: 10.1007/s12016-019-08756-7, PMID: 31267472

[25] Lemarquis A Campbell T Aranda-Guillén M Hennings V Brodin P Kämpe O . Severe COVID-19 in an APS1 patient with interferon autoantibodies treated with plasmapheresis. J Allergy Clin Immunol. (2021) 148:96–8. doi: 10.1016/j.jaci.2021.03.034, PMID: 33892926 PMC8051851

[26] Kousa AI Jahn L Zhao K Flores AE Acenas D Lederer E . Age-related epithelial defects limit thymic function and regeneration. Nat Immunol. (2024) 25:1593–1606. doi: 10.1038/s41590-024-01915-9, PMID: 39112630 PMC11362016

[27] Lemarquis AL Kousa AI Argyropoulos KV Jahn L Gipson B Pierce J . Recirculating regulatory T cells mediate thymic regeneration through amphiregulin following damage. Immunity. (2025) 58:397–411.e6. doi: 10.1016/j.immuni.2025.01.006, PMID: 39892391 PMC11932356

[28] Baba T Naka K Morishita S Komatsu N Hirao A Mukaida N . MIP-1α/CCL3-mediated maintenance of leukemia-initiating cells in the initiation process of chronic myeloid leukemia. J Exp Med. (2013) 210:2661–73. doi: 10.1084/jem.20130112, PMID: 24166712 PMC3832924

[29] Aloui C Prigent A Tariket S Sut C Fagan J Cognasse F . Levels of human platelet-derived soluble CD40 ligand depend on haplotypes of CD40LG-CD40-ITGA2. Sci Rep. (2016) 6:24715. doi: 10.1038/srep24715, PMID: 27094978 PMC4837387

[30] Granadier D Cooper K Kousa A Acenas D Lemarquis A Hernandez V . Damage-induced IL-18 stimulates thymic NK Cells limiting endogenous tissue regeneration. Nat Immunol. (2025) 26:1699–1711. doi: 10.1038/s41590-025-02270-z, PMID: 40935830 PMC12479347

[31] Ng BD Rajagopalan A Kousa AI Fischman JS Chen S Massa A . IL-18-secreting multiantigen targeting CAR T cells eliminate antigen-low myeloma in an immunocompetent mouse model. Blood. (2024) 144:171–86. doi: 10.1182/blood.2023022293, PMID: 38579288 PMC11302468

[32] Lim CK Bronson PG Varade J Behrens TW Hammarström L . STXBP6 and B3GNT6 genes are associated with selective igA deficiency. Front Genet. (2021) 12:736235. doi: 10.3389/fgene.2021.736235, PMID: 34976003 PMC8718598

[33] Ferreira RC Pan-Hammarström Q Graham RR Fontán G Lee AT Ortmann W . High-density SNP mapping of the HLA region identifies multiple independent susceptibility loci associated with selective IgA deficiency. PloS Genet. (2012) 8:e1002476. doi: 10.1371/journal.pgen.1002476, PMID: 22291608 PMC3266887

[34] Ulfarsson J Gudmundsson S Birgisdottir B Kjeld JM Jensson O . Selective serum IgA deficiency in Icelanders. Frequency, family studies and Ig levels. Acta Med Scandinavica. (1982) 211:481–7. doi: 10.1111/j.0954-6820.1982.tb01986.x, PMID: 7113764

