# Supplementary figures and images for: Serologic IL-18 increase with B-cell IL-18R loss characterizes selective IgA deficiency
Source: Front Immunol. 2026 Jan 5;16:1687720. doi: 10.3389/fimmu.2025.1687720 (PMC12812701; doi:10.3389/fimmu.2025.1687720)

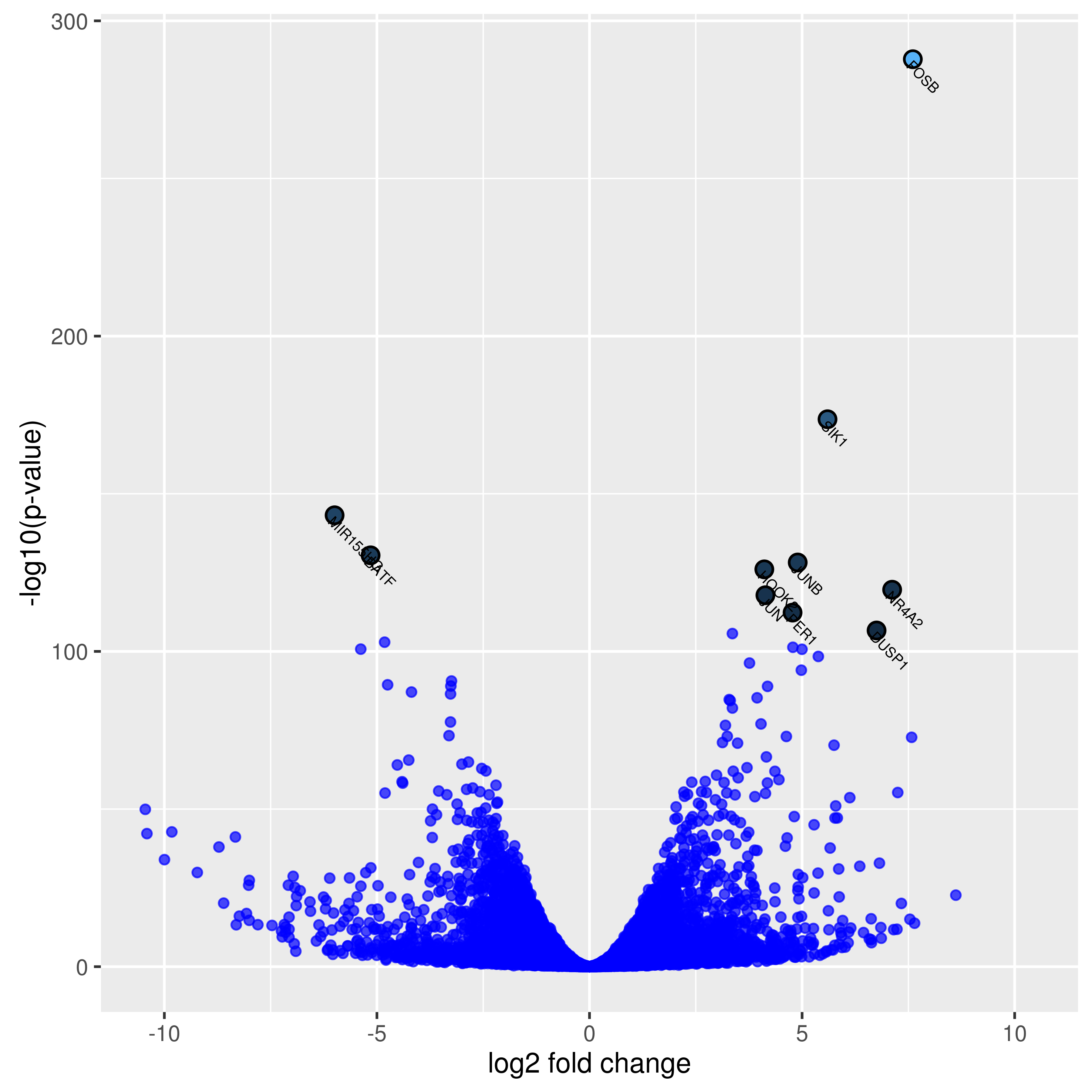

Supplement: Supplementary file 3 [file DataSheet3.zip › Paired_Differential_Expression/Paired_HC_Unstimulated-vs-HC_Stimulated/volcano.png]

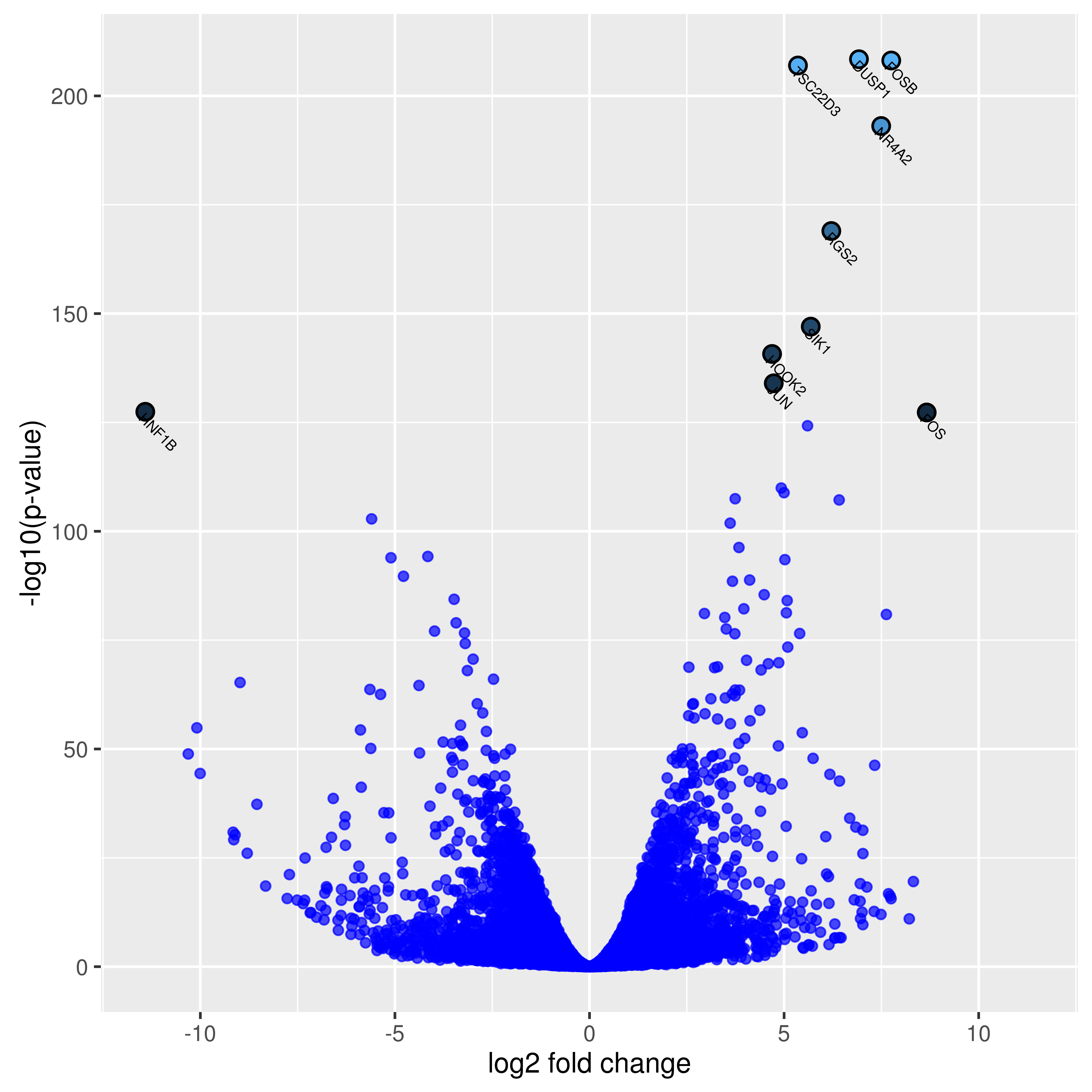

Supplement: Supplementary file 3 [file DataSheet3.zip › Paired_Differential_Expression/Paired_IgAD_Unstimulated-vs-IgAD_Stimulated/volcano.png]
